# Cord Blood FGF-21 and GDF-15 Levels Are Affected by Maternal Exposure to Moderate to Severe Anemia and Malaria

**DOI:** 10.1210/jendso/bvad120

**Published:** 2023-09-21

**Authors:** Line Hjort, Nicolai J Wewer Albrechtsen, Daniel Minja, Christine Rasmussen, Sofie Lykke Møller, John Lusingu, Thor Theander, Ib Christian Bygbjerg, Christentze Schmiegelow, Louise Groth Grunnet

**Affiliations:** Department of Obstetrics, Copenhagen University Hospital, Copenhagen 2100, Denmark; Novo Nordisk Foundation Center for Basic Metabolic Research, Metabolic Epigenetics Group, Faculty of Health and Medical Sciences, University of Copenhagen, Copenhagen 2200, Denmark; Department of Biomedical Sciences, Faculty of Health and Medical Sciences, University of Copenhagen, Copenhagen 2200, Denmark; Departments of Clinical Biochemistry, Bispebjerg and Frederiksberg Hospitals, University of Copenhagen, Copenhagen 2200, Denmark; Novo Nordisk Foundation Center for Protein Research, Faculty of Health and Medical Sciences, University of Copenhagen, Copenhagen 2200, Denmark; National Institute for Medical Research, Tanga Center, Tanga 5004, Tanzania; Department of Biomedical Sciences, Faculty of Health and Medical Sciences, University of Copenhagen, Copenhagen 2200, Denmark; Departments of Clinical Biochemistry, Bispebjerg and Frederiksberg Hospitals, University of Copenhagen, Copenhagen 2200, Denmark; Department of Public Health, Section of Global Health, University of Copenhagen, Copenhagen 2200, Denmark; National Institute for Medical Research, Tanga Center, Tanga 5004, Tanzania; Department of Immunology and Microbiology, Centre for Medical Parasitology, University of Copenhagen, Copenhagen 2200, Denmark; Department of Infectious Diseases, Copenhagen University Hospital, Copenhagen 2100, Denmark; Department of Public Health, Section of Global Health, University of Copenhagen, Copenhagen 2200, Denmark; Department of Immunology and Microbiology, Centre for Medical Parasitology, University of Copenhagen, Copenhagen 2200, Denmark; Department of Infectious Diseases, Copenhagen University Hospital, Copenhagen 2100, Denmark; Clinical Research, Steno Diabetes Center Copenhagen, Herlev 2730, Denmark

**Keywords:** FGF-21, GDF-15, pregnancy, offspring, moderate to severe anemia, malaria

## Abstract

**Context:**

Anemia and malaria are global health problems affecting >50% of pregnant women in sub-Saharan Africa and are associated with intrauterine growth restriction. The hormones fibroblast growth factor 21 (FGF-21) and growth differentiation factor 15 (GDF-15) are involved in metabolic regulation and are expressed in the placenta. No studies exist on FGF-21 and GDF-15 responses to exposures of malaria and anemia in pregnancy.

**Objective and Methods:**

Using a prospective, longitudinal pregnancy and birth cohort of women with an average age of 26 years from a rural region in northeastern Tanzania, we examined if FGF-21 and GDF-15 levels in maternal blood at week 33 ± 2 (*n* = 301) and in cord blood at birth (*n* = 353), were associated with anemia and malaria exposure at different time points in pregnancy and with neonatal anthropometry.

**Results:**

Among mothers at gestation week 33 ± 2, lower FGF-21 levels were observed after exposure to malaria in the first trimester, but not anemia, whereas GDF-15 levels at week 33 ± 2 were not associated with malaria nor anemia. In cord blood, moderate to severe anemia at any time point in pregnancy was associated with higher levels of FGF-21, whereas malaria exposure in the third trimester was associated with lower FGF-21 levels in cord blood. Negative associations were observed between cord blood FGF-21 and GDF-15 levels and neonatal skinfold thicknesses and birthweight.

**Conclusion:**

Our results suggest that moderate to severe anemia throughout pregnancy associates with higher FGF-21 levels, and malaria in last trimester associates with lower FGF-21 levels, in the neonates, thereby potentially affecting the future cardiometabolic health of the child.

Anemia is a global health problem affecting 36% to 60% of pregnant women in low- and middle-income countries (LMICs) [1‐3]. In sub-Saharan Africa (SSA), major risk factors for anemia in pregnancy are iron deficiency and malaria infections, especially *Plasmodium falciparum* [3, 4]. Maternal iron deficiency compromises the fetal iron supply, and iron deficiency anemia is associated with increased risk of being born with low birth weight (LBW) [5]. Both moderate to severe maternal anemia and malaria in pregnancy have been linked with intrauterine growth restriction (IUGR) and LBW [6‐9]. Furthermore, both malaria and anemia have been associated with adverse development of the placenta [10‐14], including interaction with the insulin-like growth factor axis that is important for fetal and placental growth [4, 15]. Hence, both anemia and malaria in pregnancy may have detrimental consequences on pregnancy outcome, including LBW, which in itself is associated with an increased risk of a range of noncommunicable diseases (NCDs) later in life, including obesity and cardiometabolic diseases [16]. Currently, SSA countries like Tanzania are experiencing a fast epidemiological transition, from a population affected by anemia and infectious diseases, toward being affected by NCDs within one generation [17, 18]. However, the ways in which anemia and malaria may influence offspring susceptibility to NCDs remain poorly understood.

Fibroblast growth factor 21 (FGF-21), a circulating hormone secreted from the liver, and in pregnancy also by the placenta [19], has been found to be increased in subjects with type 2 diabetes [20]. In mice, FGF-21 has advantageous effects on glucose and lipid metabolism, whereas FGF-21 in humans is thought to improve lipid profiles with no reduction in glucose concentrations [21]. FGF-21 analogues are in clinical trials for treatment of severe liver diseases (nonalcoholic steatohepatitis) [22]. Maternal FGF-21 plasma concentrations are increased from the first to third trimester of pregnancy, and maternal FGF-21 concentrations have been positively correlated with body mass index (BMI) and adiposity but not lean mass or glucose homeostasis [19].

The function of growth differentiation factor 15 (GDF-15), also called macrophage inhibitory cytokine-1, is not fully understood, but it has been shown to play several important roles in regulating inflammatory pathways, and its levels are increased under stress conditions and in cancer [23]. GDF-15 is expressed to a very low degree in most somatic tissues, but it is abundant in placental and maternal circulation in pregnancy [24]. In the third trimester, it is 200-fold higher compared to the nonpregnant state [24], and it is speculated that this reduces exposure to teratogens in pregnancy [25]. Furthermore, both circulating FGF-21 [26] and circulating GDF-15 are increased in pre-eclampsia [23]. Knowledge about the role of GDF-15 in body weight homeostasis and pregnancy is limited but currently growing.

Studies of FGF-21 and GDF-15 in pregnancy are sparse and the responses of these hormones to exposures like malaria or anemia in pregnancy are not known. In the present exploratory study, we hypothesized, based on the inflammatory state of malaria and anemia, that anemia and/or malaria during pregnancy would be associated with increased FGF-21 and GDF-15 levels during pregnancy and in the cord blood, which potentially could be part of the underlying mechanisms behind an increased risk of cardiometabolic diseases later in life caused by an adverse intrauterine environment. To investigate if maternal anemia or malaria in pregnancy affected maternal and/or newborn FGF-21 and GDF-15 levels, we measured the circulating plasma levels of FGF-21 and GDF-15 in 301 fasting maternal venous plasma samples at gestational age (GA) of 33 ± 2 weeks, and in 353 cord blood plasma samples collected at birth. Furthermore, we examined if the FGF-21 and GDF-15 levels were associated with neonatal body compositional outcome at birth.

## Methodology

### Study Design and Population

The study cohort is a population based prospective study established in the Korogwe and Handeni districts, Tanga region, Tanzania, and is titled FOETALforNCD (foetal exposure and epidemiological transition: the role of anemia in early life for non-communicable diseases in later life) [27]. The field study was conducted in a rural region of North-East Tanzania, from July 2014 to December 2016. The central field site was the maternity ward and the reproductive and child health clinic (RCH clinic) at Magunga Korogwe District Hospital.

**Figure 1.**
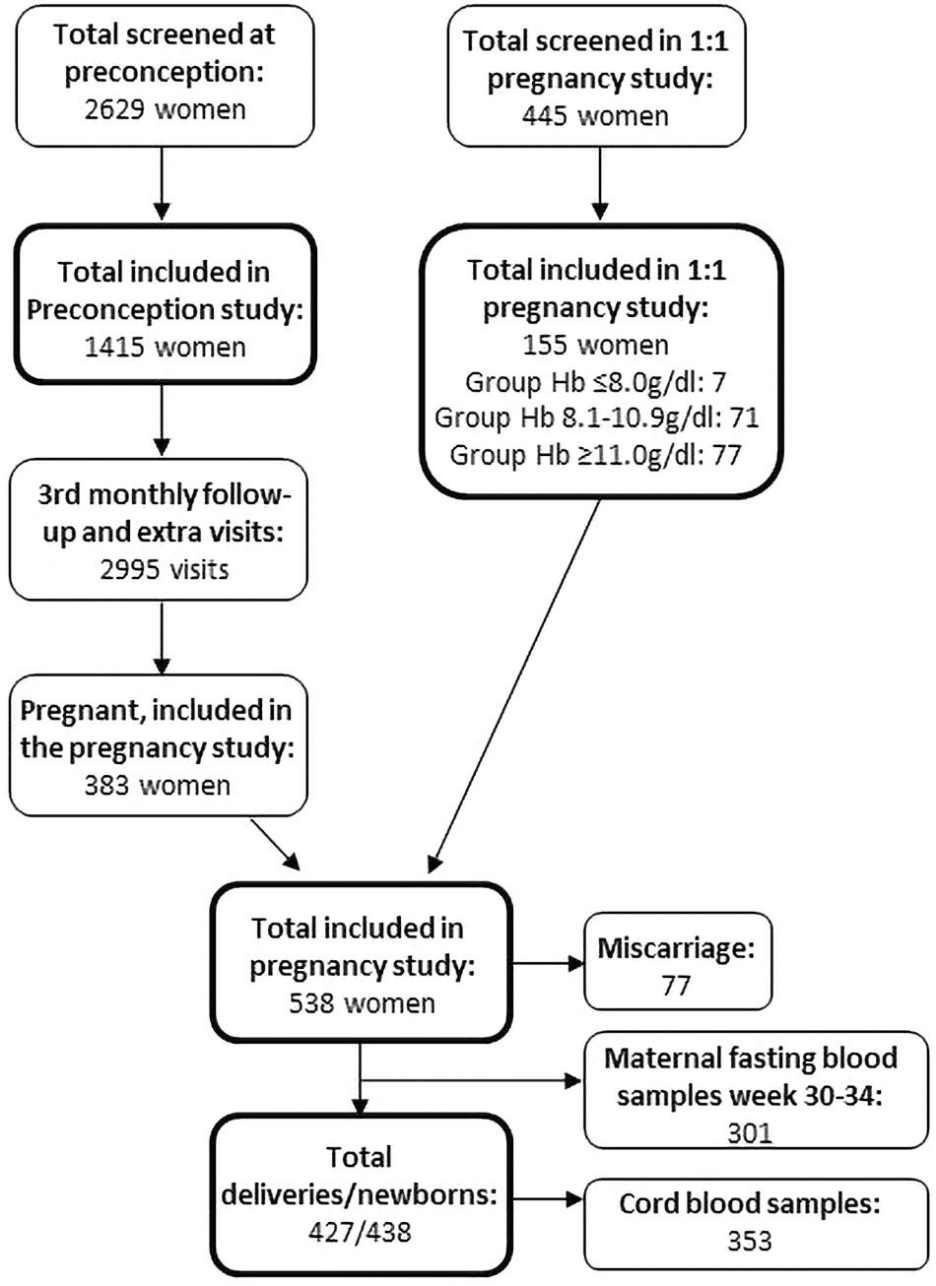
Overview of enrollment of women in the 2 inclusion arms of the FOETALforNCD cohort study: at preconception or in first trimester (1:1 pregnancy study). Numbers of maternal fasting samples at week 33 ± 2 and cord blood samples reflect the number of plasma samples that were available for the FGF-21 and GDF-15 measurements.

The study design has been described previously in detail [27]. In brief, all participants were recruited prior to conception or in early pregnancy (Fig. 1) and followed throughout pregnancy until birth and 1 month postnatally. The preconception study population was supplemented with screening of women in early pregnancy (GA of ≤14 weeks), on a 1:1 basis with a hemoglobin (Hb) ≤8 g/dL (severe anemia) or Hb 8.1 to 10.9 g/dL (mild to moderate anemia): Hb ≥11 g/dL (nonanemic), to secure a sufficiently powered anemic vs nonanemic sample size, as described previously [27]. In total, 538 women participated in the pregnancy study and 427 in the birth study (Fig. 1). Data collection included maternal anthropometrics; sociodemographics; blood samples; screening for diabetes, hypertension, and malaria; ultrasound in each trimester; and cord blood samples. At the time of delivery, measurements of neonatal body composition—newborn weight, length, head circumference, abdominal circumference, mid-upper arm circumference (MUAC), and skinfold thickness of biceps, triceps, subscapular, and thigh—were taken. Birth weight Z-scores were estimated using a reference chart from Tanzania [28‐31].

### Ethical Considerations

The study received ethical approval from the Tanzania Medical Research Coordinating Committee of the National Institute for Medical Research (MRCC) (reference number: NIMR/HQ/R.8a/Vol. IX/1717). Written informed consent or thumbprint (for women who were illiterate) was obtained prior to enrollment. All study procedures were performed according to good clinical and laboratory practices and the Declaration of Helsinki. All participants were treated according to Tanzanian national guidelines and the project assisted all participants in obtaining the best local medical care available if disease was diagnosed. The data sharing follows the local NatHREC requirements.

### Point-of-Care Diagnostics and Treatment

At the study site, point-of-care diagnostics were performed on the venous blood, including Hb level and malaria infection at all visits. Hemoglobin levels were measured using HemoCue 301 Hb analyzer (HemoCue AB). According to World Health Organization (WHO) definitions, preconception anemia was defined as Hb < 12.0 g/dL and anemia in pregnancy as Hb < 11.0 g/dL. All women were offered daily iron and folic acid supplementation throughout pregnancy (200 mg ferrous sulfate [∼43 mg elemental iron] and 400 µg folate per day [Ferrolic–LF^®^, Laboratory and Allied LTD, Mombasa, Kenya]). If anemic, supplementation was increased to 2 to 3 combination tablets of iron-folic acid per day or changed to Hemovit^®^ multivitamin syrup (200 mg ferrous sulfate, 0.5 mg B6, 50 µg B12, 1.5 mg folic acid and 2.33 mg zinc sulfate per 5 mL; Shelys Pharmaceuticals) 10 mL 2 to 3 times daily depending on severity of the anemia.

Women were screened for malaria using malaria rapid diagnostic test kit (ParaHIT, Span Diagnostics or CareStart Malaria Pf, ACCESS BIO). Microscopy were performed on all samples, and PCR on a subset, to confirm malaria positivity [32]. Malaria patients were offered oral artemether-lumefantrine (Lumartem 20 mg/120 mg, Cipla Ltd), quinine, or artesunate injections depending on severity and GA [32]. Fasting glucose levels were measured for gestational diabetes mellitus screening at week 33 ± 2 using Hemocue 301+ Glu analyzer (HemoCue AB) [33].

### Blood Sample Collection and Processing

Venous and cord blood was collected in EDTA-coated tubes and transported at 2 to 8 °C to the National Institute for Medical Research (NIMR) Korogwe Research Field Station Laboratory for further processing within 4 hours of collection [27]. For data analyses, Hb levels were measured using Sysmex KX-21N hematological analyzer (Sysmex Corporation) at all contacts with the woman during pregnancy [27]. All samples were stored at −80 °C and later shipped on dry ice to the Department of Clinical Biochemistry, Copenhagen University Hospital/Rigshospitalet, Denmark, for measurements of FGF-21 and GDF-15 concentrations.

In total, 301 maternal fasting plasma samples from pregnancy week 33 ± 2, and 353 cord blood samples were available for the present study. Of these, there were 255 matching maternal and cord blood pairs. Measurement of FGF-21 and GDF-15 was performed using evaluated immunoassays (R&D, Catalog number DF2100 and DGD150). FGF-21 levels were successfully measured in 298 maternal samples and 263 cord blood samples, and GDF-15 in 291 maternal samples and in 324 cord blood samples (coefficients of variation < 20). The remaining failed primarily due to low detection rates caused by hemolysis.

### Exposure Groups

For data analysis, maternal anemia and malaria occurring at any time point during pregnancy were treated as the exposure variables, while maternal and cord blood FGF-21 and GDF-15 were the primary outcomes.

A meta-analysis of 12 studies with a total of 341 823 mother-child dyads demonstrated that Hb levels below 9 or 8 g/dL, but not Hb levels below 10 or 11 g/dL, gave rise to small for gestational age (SGA) newborns, and hence defined Hb < 9.0 g/dL as moderate to severe anemia [8]. Furthermore, Dewey et al have demonstrated a U-shaped relationship between Hb level and pregnancy outcomes, with Hb levels in the mid-range having the least consequences: stillbirth was associated with Hb levels ≤9 g/dL or ≥18 g/dL; preterm birth associated with Hb levels ≤9 g/dL or ≥17 g/dL; and SGA associated with Hb levels <9 g/dL or ≥13 g/dL [7]. Based on the above studies, we therefore in the present study defined any anemia in pregnancy as Hb < 11.0 g/dL, and moderate to severe anemia as Hb < 9.0 g/dL.

Scanlon et al demonstrated in a retrospective cohort of 173 031 pregnant women that the odds of SGA were increased with higher Hb levels. They defined very high Hb level as 14.9 g/dL in the first trimester and as 14.4 g/dL at 18 weeks of gestations, both equivalent to 3 SDs above the median Hb level [34]. To limit the risk of bias to the results being introduced by the potential effects from a higher Hb level in pregnancy, we therefore defined the upper cutoff as the average Hb level + 3SDs in the second trimester (average gestational age: week 20 + 4), which in our study was 13.98 g/dL. Hence, participants with an Hb > 14.0 g/dL at any point during pregnancy (*n* = 26) were excluded from the exposure analyses.

Malaria is especially detrimental among paucigravidae women [6, 9, 35], and we therefore examined the association between maternal malaria exposure and cord blood FGF-21 and GDF-15 levels among only first and second gravidae women (*n* = 102, out of 301). When examining the association between maternal malaria exposure and maternal blood FGF-21 and GDF-15 levels, all women were included in the analyses (*n* = 301).

Data were analyzed according to the timing in pregnancy where the exposures had occurred, by stratifying by: first trimester (week 0-14), early second trimester (week 15-22), late second trimester (week 23-28), and third trimester (week 29 to birth), as performed previously [13, 14]. The control group was defined as women never having malaria or never having any type of anemia (never Hb < 11.0 g/dL) before the specific time point of FGF-21 or GDF-15 measurement.

### Statistics

Statistical analysis was conducted with RStudio software (version 4.1.0) and SAS Enterprise Guide (version 7.1). Graphic illustrations were developed with GraphPad Prism (version 9.0). Categorical variables are presented in the form of counts (percentage). Continuous variables are presented with mean (±SD) for parametric distributions and with median (interquartile range [IQR]) for nonparametric distributions. Hormone level differences between exposure groups were evaluated with Student *t* test and Wilcoxon rank score for continuous parametric and nonparametric distributed variables, respectively, and with χ^2^ test for categorical variables. Correlation analyses were performed via Spearman rank analysis. All tests were 2-tailed and *P* values <.05 were considered significant.

Adjusted linear regression modeling was performed to estimate associations between cord blood hormone levels and newborn body size parameters. Two models were applied: model 1 included adjusting for gestational age at birth, offspring sex, and exposure to anemia and/or malaria in pregnancy. Model 2 included all the covariates from Model 1 plus maternal age, parity, hypertension (including pre-eclampsia) in pregnancy, maternal HIV status, maternal education level, and maternal MUAC in first trimester. Assumptions of equal variance and normally distributed residuals were visualized in QQ plots and histograms.

## Results

### Clinical Characteristics of Mothers and Newborns

The women were on average 26 years of age and the majority were pregnant for the third or more time (71%). In the first trimester, the median maternal weight was 55.2 kg with an average BMI of 22.8 m^2^/kg (Table 1). The majority of the enrolled women were of either Sambaa or Zigua ethnicity (70%). In total 2.5% were HIV positive.

**Table 1.** Maternal, pregnancy, birth, and newborn characteristics

Maternal characteristics		*n*
Age (years)	26 (22-34)	369
Ethnicity		374
Sambaa	138 (37%)	
Zigua	126 (34%)	
Others*^a^*	110 (29%)	
Parity		374
0	53 (14%)	
1	76 (20%)	
2 or more	245 (66%)	
Gravidity		374
1	41 (11%)	
2	67 (18%)	
3 or more	266 (71%)	
HIV seropositive	10 (2.7%)	374
*First trimester maternal clinical characteristics*		
Height (cm)	155 ± 5.8	374
Weight (kg)	55.2 (48.8-63.3)	368
BMI (kg/m^2^)	22.8 (20.5-25.7)	368
Mid-upper arm circumference (cm)	27.4 (25.3-30.1)	370
Skinfold thickness (mm)	15.4 (11.2-21.0)	350
Systolic blood pressure (mmHg)	112 ± 11	371
Diastolic blood pressure (mmHg)	71 ± 9	371
*Pregnancy and birth characteristics*		
Any anemia (Hb < 11.0)	292 (78%)	374
Moderate-severe anemia (Hb < 9.0)	70 (19%)	374
Hb > 14.0	26 (7%)	374
Hb pre-pregnancy (mmol/L)	12.3 (11.4-13.2)	172
Hb in first trimester (mmol/L)	11.7 (10.8-12.4)	371
Hb in third trimester (mmol/L)	10.7 ± 1.3	301
Hb at delivery (mmol/L)	11.2 ± 1.5	372
Fasting glucose in third trimester (mmol/L)	4.86 ± 0.74	301
Malaria positive during pregnancy	150 (40%)	374
Pre-eclampsia	7 (2%)	363
Gestational age at delivery (days)	281 (281-287)	374
Preterm delivery (GA < 37 weeks)	17 (4.6%)	374
Sex (female)	192 (51.3%)	374
Birthweight (g)	3022 ± 499	373
Placental weight (g)	454 ± 105	364
Hb in cord blood (mmol/L)	14.2 ± 1.7	334

Data are presented as mean (±SD), median (IQR) or *n* (%).

Abbreviations: BMI, body mass index; GA, gestational age; Hb, hemoglobin.

a All other tribe groups include tribes with <10% prevalence.

The cumulative prevalence during pregnancy of any degree of anemia (Hb < 11.0) was 78%, whereas for moderate to severe anemia (Hb < 9.0) it was 19% (Table 1). Furthermore, 40% of the participants were infected with malaria at least once during their pregnancy, and 2% were diagnosed with pre-eclampsia (Table 1). Average Hb levels decreased slightly from 11.7 g/dL in the first trimester to 11.2 g/dL at delivery (Table 1). The average GA at birth was 281 days (Table 1) corresponding to 40.1 weeks of gestation.

### Maternal FGF-21 and GDF-15 Levels in Response to Anemia or Malaria Exposure in Pregnancy

**Figure 2.**
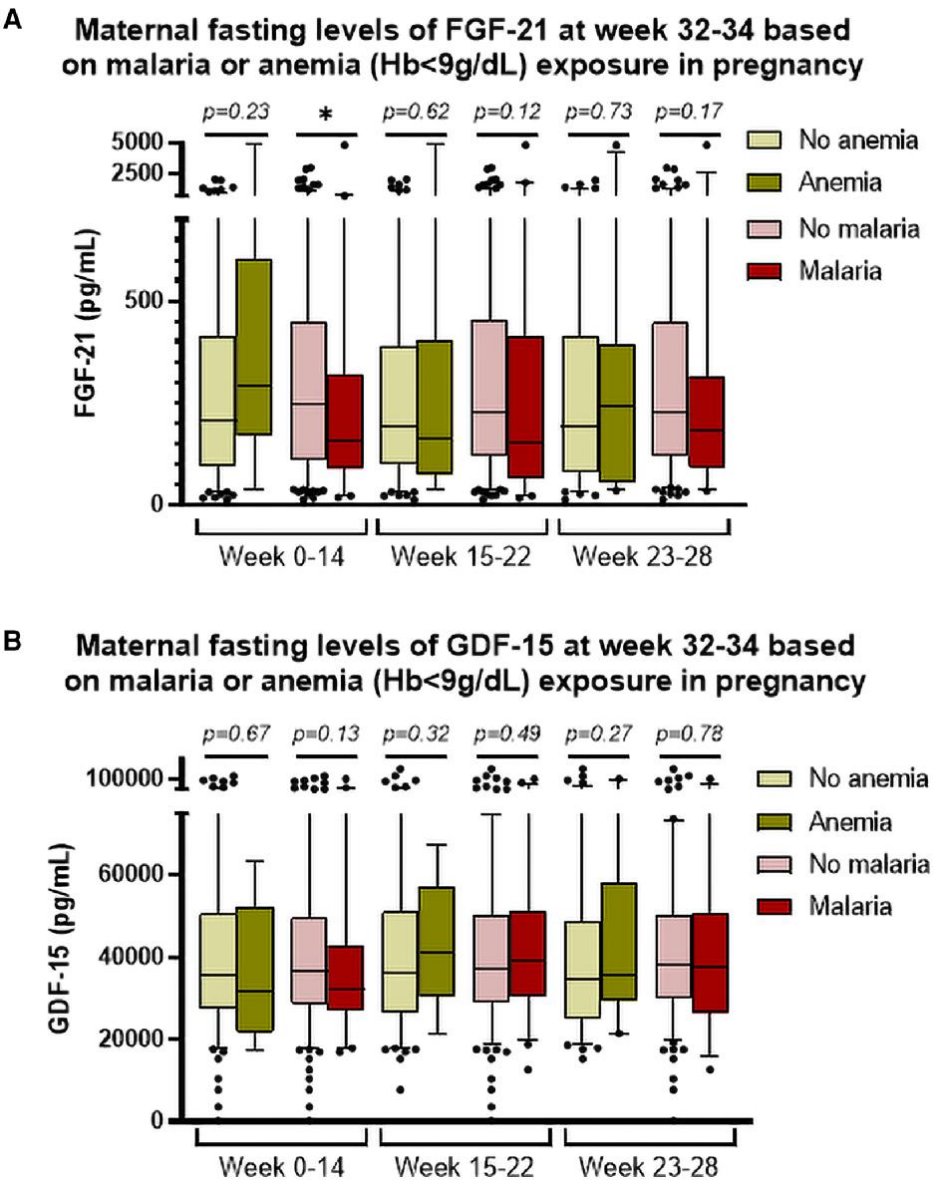
Maternal fasting levels at weeks 33 ± 2 of A) FGF-21, and B) GDF-15, in response to moderate to severe anemia (green columns) or exposure to malaria (red columns) during the first (weeks 0-14), second (weeks 15-22), and late second/early third (weeks 23-28) trimester. Data presented as median with 95% CI. *: *P* < 0.01. Sample size for anemia exposure analyses: *n* = 9-10 exposed vs 152-158 unexposed (week 0-14), *n* = 16-17 exposed vs 120-126 unexposed (week 15-22), *n* = 22 exposed vs 94-98 unexposed (week 23-28). Sample size for malaria exposure analyses: *n* = 41-42 exposed vs 184-188 unexposed (week 0-14), *n* = 46 exposed vs 191-197 unexposed (week 15-22), *n* = 33 exposed vs 171-177 unexposed (week 23-28).

FGF-21 levels in the third trimester were ∼40% lower in mothers who had been exposed to malaria during the first trimester compared to those without malaria exposure (Fig. 2A). The same tendency was observed in mothers exposed to malaria in the second and third trimester, but this did not reach statistical significance (*P* ≥ .12) (Fig. 2A). In contrast, moderate to severe anemia (Hb < 9.0 g/dL) at any time during pregnancy did not affect maternal circulating FGF-21 levels in the third trimester (week 33 ± 2) (Fig. 2A). Maternal GDF-15 levels were not affected by either moderate to severe anemia or malaria in pregnancy (Fig. 2B).

### Cord Blood FGF-21 Levels Are Affected by Both Maternal Anemia and Malaria in Pregnancy but in Opposite Directions

**Figure 3.**
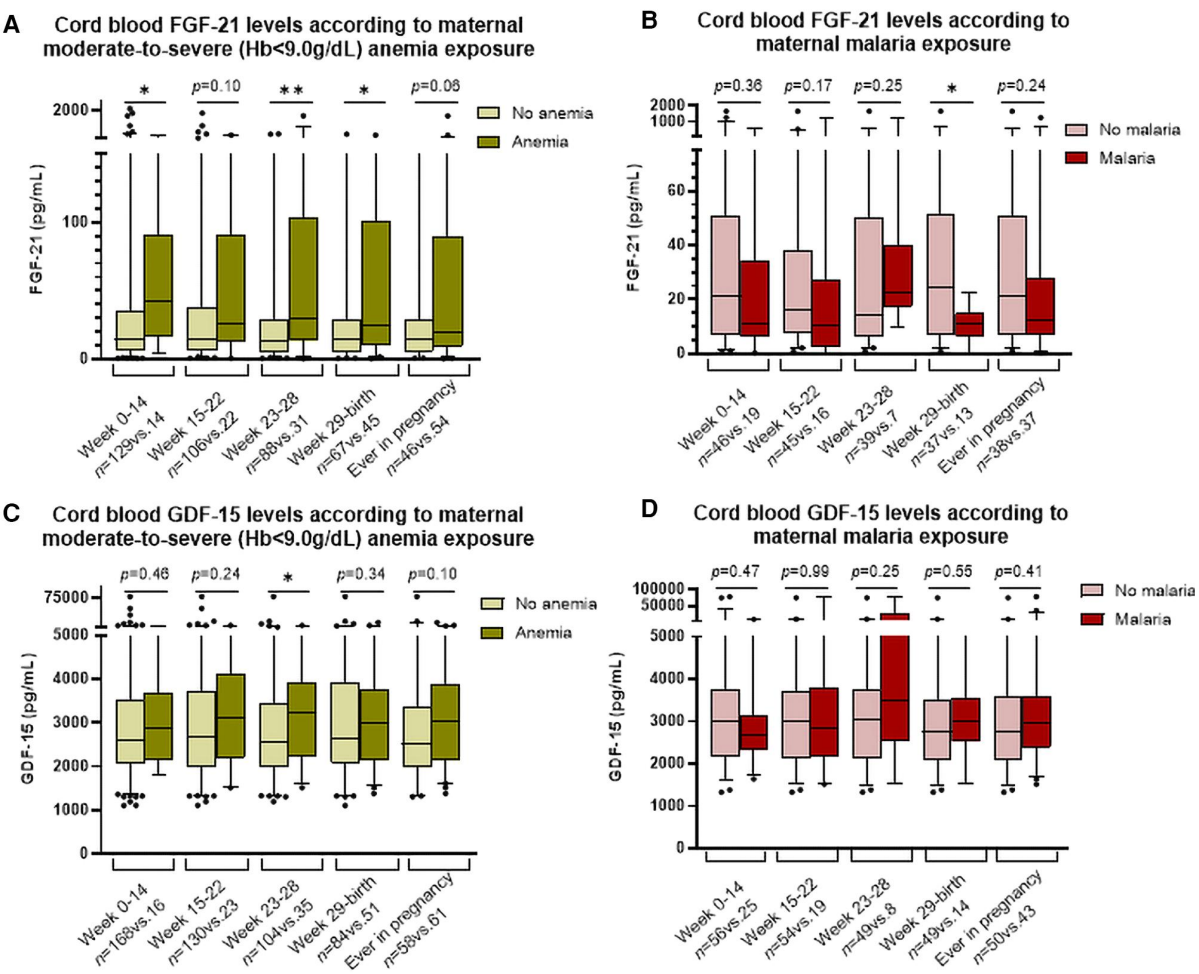
Cord blood plasma levels of A, B) FGF-21 and C, D) GDF-15, in response to maternal exposure to moderate to severe anemia (green columns) and to malaria (red columns) during pregnancy in weeks 0-14, 15-22, 23-28, and from week 29 to birth. Data presented as median with 95% CI. *: *P* < 0.01, **: *P* < 0.001.

Moderate to severe anemia (Hb < 9.0 g/dL) in week 0-14, week 23-28, or from week 28 to birth, was associated with ∼3-fold increased FGF-21 levels in the cord blood (*P* ≤ .02, Fig. 3A). Similar tendencies were observed for weeks 15 to 22, although without reaching statistical significance (*P* = .10) (Fig. 3A). In contrast to anemia, maternal malaria infection in the third trimester was associated with lower cord blood FGF-21 levels (*P* = .04, Fig. 3B). Similar patterns were observed for malaria during the first trimester, and in early and late second trimesters but were not statistically significant (*P* = .36, *P* = .17, and *P* = .25, respectively).

### Cord Blood GDF-15 Levels Are Affected by Maternal Anemia, but Not Malaria, in Pregnancy

GDF-15 levels in cord blood were increased in pregnancies where the mother was exposed to moderate to severe anemia in the late second trimester (week 23-28), and a similar pattern seemed to be present for the other time points in pregnancy, although not statistically significant (*P =* .10-.46) (Fig. 3C). Furthermore, the cord blood GDF-15 levels were similar between women exposed to malaria during pregnancy and controls (Fig. 3D).

### Moderate to Severe Anemia, but Not Mild Anemia, at Preconception and in the First Half of Pregnancy Is Associated With Cord Blood FGF-21 Levels

**Figure 4.**
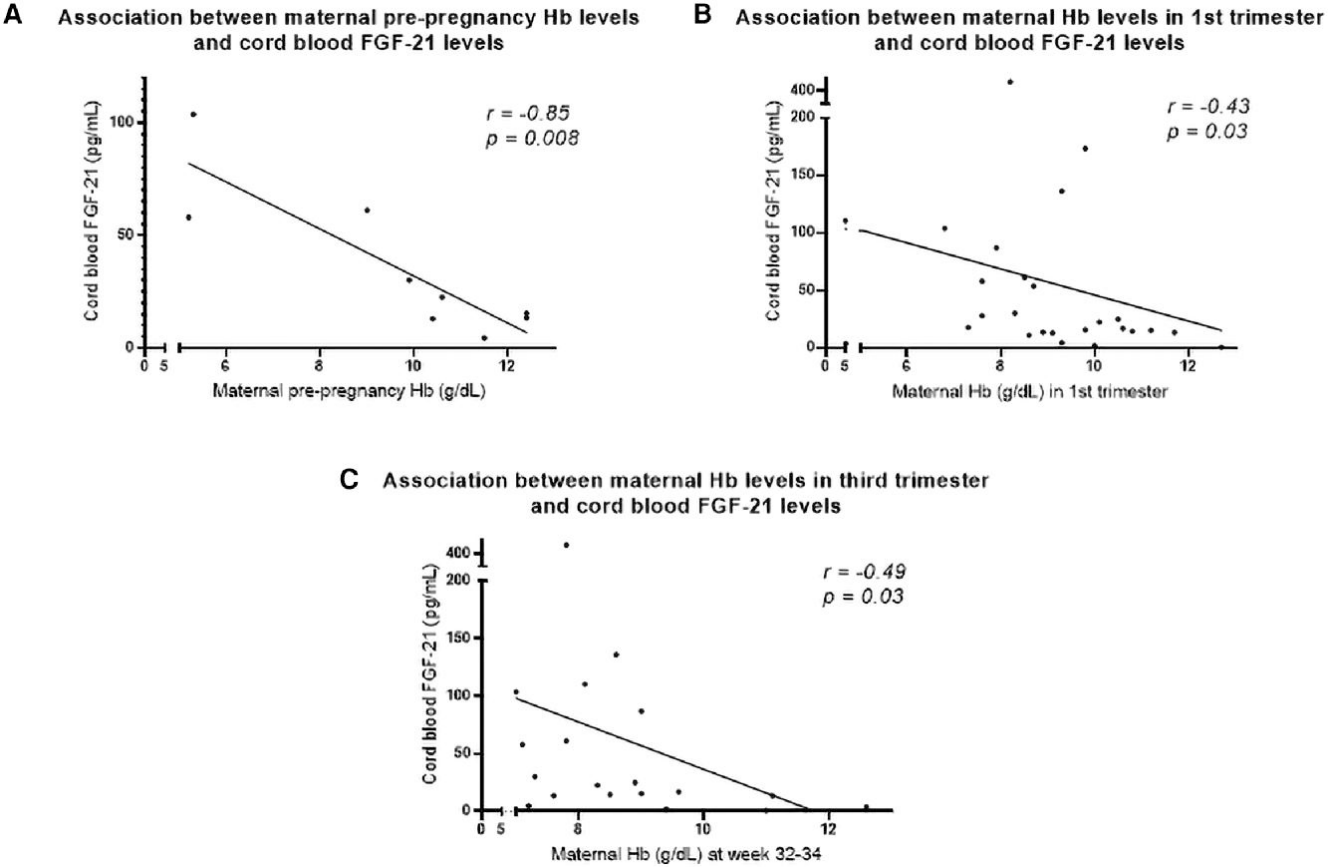
Cord blood FGF-21 plasma levels in response to maternal Hb levels at A) pre-pregnancy, B) first trimester, and C) third trimester.

To further explore whether a specific degree and timing of anemia was linked to the observed changes in cord blood FGF-21 levels, we performed correlation analysis between cord blood FGF-21 and maternal Hb levels measured at pre-pregnancy, in the first trimester and the third trimester. Interestingly, for women who were exposed to moderate to severe anemia during the first half of their pregnancy (before week 22), we found significant negative associations between cord blood FGF-21 and maternal Hb levels, at both preconception, in the first trimester and in the third trimester, (*r* ≥ −0.85, *P* = .008, *r* ≥ −0.43, *P* = .03, *r* ≥ −0.49, *P* = .03, Fig. 4), with the strongest association observed pre-pregnancy. For pregnancies exposed to any degree of anemia (Hb < 11.0 g/dL) before week 22, same patterns were observed, but to a lower degree (*r* ≥ −0.18, *P* ≤ .05, results not shown). Similar trends were observed among women exposed to moderate to severe anemia in the second half of the pregnancy (after week 22), albeit not statistically significant (*P* ≥ .06, results not shown).

### Association Between FGF-21 and GDF-15 Levels and Phenotypes

To examine if the FGF-21 and GDF-15 levels in mother and child were associated with maternal characteristics or neonatal outcome at birth, we first performed simple correlation analyses (Table 2). We observed that maternal FGF-21 and GDF-15 levels at weeks 33 ± 2 were positively associated with higher GA at the time of the blood sampling (*P* ≤ .03). Furthermore, maternal GDF-15 levels were negatively associated with maternal BMI and MUAC in the first trimester (*P* ≤ .005). Neither maternal FGF-21 nor GDF-15 levels were associated with neonatal body composition outcomes; however, we found that both FGF-21 and GDF-15 cord blood levels were negatively associated with the birth weight Z-score and the supra-iliac skinfold thickness (Table 2). Finally, we found that maternal fasting FGF-21 and GDF-15 levels in weeks 33 ± 2 were positively associated with the cord blood levels of FGF-21 (*r* = 0.16, *P =* .02), and GDF-15 (*r* = 0.28, *P <* .001), respectively.

**Table 2.** Associations between maternal and cord blood FGF-21 and GDF-15 levels and clinical characteristics

	Maternal fasting levels in week 33 ± 2						Cord blood levels at birth					
	FGF-21 (pg/mL)			GDF-15 (pg/mL)			FGF-21 (pg/mL)			GDF-15 (pg/mL)		
	r	*P value*	*n*	r	*P value*	*n*	r	*P value*	*n*	r	*P value*	*n*
Maternal age	**0**.**15**	***0***.***009***	**294**	−0.10	*0*.*10*	287	0.02	*0*.*76*	260	−0.01	*0*.*85*	321
BMI first trimester	0.08	*0*.*16*	294	**−0**.**14**	***0***.***02***	**287**	−0.04	*0*.*53*	259	0.06	*0*.*26*	318
MUAC first trimester	**0**.**12**	***0***.***05***	**294**	**−0**.**16**	***0***.***006***	**288**	0.001	*0*.*99*	259	0.06	*0*.*28*	*321*
GA at sample collection	**0**.**17**	***0***.***005***	**279**	**0**.**19**	***0***.***002***	**272**	**0**.**15**	***0***.***02***	**247**	−0.01	*0*.*85*	303
Birthweight	0.08	*0*.*17*	298	0.03	*0*.*62*	291	−0.10	*0*.*09*	262	**−0**.**11**	***0***.***05***	**323**
Birthweight Z-score	0.06	*0*.*33*	293	0.09	*0*.*13*	286	**−0**.**16**	***0***.***01***	**258**	**−0**.**12**	***0***.***04***	**319**
ST Triceps	0.04	*0*.*52*	278	0.02	*0*.*80*	271	−0.10	*0*.*13*	244	−0.10	*0*.*09*	300
ST Biceps	0.08	*0*.*16*	278	−0.04	*0*.*49*	271	**−0**.**14**	***0***.***03***	**244**	−0.08	*0*.*16*	300
ST Subscapular	−0.01	*0*.*86*	278	−0.01	*0*.*86*	271	−0.08	*0*.*23*	244	−0.01	*0*.*88*	300
ST Supra-iliac	−0.01	*0*.*91*	278	−0.02	*0*.*80*	271	**−0**.**14**	***0***.***03***	**244**	**−0**.**13**	***0***.***03***	**300**

Correlation analyses performed by Spearman rank analysis, and data are presented as r-coefficient and *P* value.

Abbreviations: GA, gestational age (days); ST, skinfold thickness.

We further investigated the significant correlations observed between cord blood FGF-21 and GDF-15 levels and newborn body size, using multiple linear regression modeling (Table 3). When adjusting only for gestational age at birth, offspring sex, and anemia and/or malaria exposure in pregnancy, the negative association between cord blood GDF-15 levels and newborn birthweight remained significant (model 1, Table 3). However, when also adjusting for the maternal factors of age, MUAC in first trimester, hypertension in pregnancy, parity, HIV status, and educational level, only the negative association between cord blood GDF-15 levels and birth weight Z-score remained statistically significant (model 2, Table 3).

**Table 3.** Associations between cord blood FGF-21 and GDF-15 levels and newborn body size

	Model 1				Model 2			
	FGF-21 (pg/mL)		GDF-15 (pg/mL)		FGF-21 (pg/mL)		GDF-15 (pg/mL)	
	β	*P value*	β	*P value*	β	*P value*	β	*P value*
*Neonatal body composition*								
Birth weight (g)	−0.09314	.*36*	−0.00574	.***048***	−0.04846	.*62*	−0.00411	.*15*
Birth weight Z-score*^a^*	−0.00031749	.27	−0.00002488	.***002***	−0.00018286	.*51*	−0.00001838	.***02***
ST Triceps (mm)	−0.0008121	.*33*	−0.00001056	.*14*	−0.00008818	.*29*	−0.00000984	.*16*
ST Biceps (mm)	−0.00008108	.*27*	−0.00001039	.*12*	−0.00006490	.*39*	−0.00000953	.*15*
ST Subscapular (mm)	−0.0001407	.*14*	−0.00000683	.*41*	−0.00016943	.*08*	−0.00000532	.*51*
ST Supra-iliac (mm)	−0.00018256	.*19*	−0.00001935	.*10*	−0.00018496	.*19*	−0.00001760	.*12*

Changes in newborn body size variables with 1 unit increase in cord blood FGF-21 or GDF-15 levels (pg/mL), presented as β estimates and *P* value.

Model 1: Adjusted for: anemia and/or malaria exposure in pregnancy, sex, gestational age at birth.

Model 2: Model 1 + age, parity, hypertension (including pre-eclampsia) in pregnancy, maternal HIV status, maternal education level, maternal MUAC in first trimester.

Abbreviation: ST, skinfold thickness.

a Birth weight Z-score models are without adjusting for sex, since the Z-score estimate already includes adjustment for sex.

## Discussion

This study is to the best of our knowledge the first to investigate FGF-21 and GDF-15 levels in pregnant women and in cord blood in a rural sub-Saharan African population setting, as well as to assess the association between anemia and/or malaria with FGF-21 and GDF-15 levels in the mother and the offspring. We observed a modest decrease in maternal circulating levels of FGF-21 in response to malaria exposure in first trimester, but not to anemia exposure, and no impact on GDF-15 levels in response to either anemia or malaria. Interestingly, we found that moderate to severe anemia, particularly in the first half of pregnancy, was associated with increased levels of FGF-21 levels in cord blood. In contrast, malaria exposure in the third trimester appeared to have opposite impact, resulting in lower FGF-21 levels in cord blood. Finally, significant but relatively weak negative correlations were observed between cord blood FGF-21 and GDF-15 levels and neonatal skinfold thicknesses and Z-score.

### Maternal FGF-21 and GDF-levels After Anemia and Malaria in Pregnancy

Our results show that maternal malaria in pregnancy, but not anemia, may lower the woman's circulating FGF-21 levels around the third trimester. We have previously shown that malaria in pregnancy, and especially in the first trimester, results in smaller, hypo-vascularized placentas [13]. Since the placenta is producing FGF-21 increasingly in correlation to placental growth [19], we can speculate that the decreased FGF-21 levels observed here, are explained by the women having smaller placentas and hence less expression of FGF-21. In addition, Sutton et al have previously shown that maternal FGF-21 plasma concentrations in pregnancy were not linked to changes in maternal energy stores (reflected by weight gain and body composition), but rather inversely associated to changes in fasting glucose levels [19]. Those findings may suggest that FGF-21 acts as a signal of maternal nutrient status in pregnancy. In our study, we could confirm that GA was positively linked to maternal FGF-21 levels, but we found no association with fasting plasma glucose levels. This might reflect that the pregnant women in our study were younger and had a lower BMI, than the women in the North American study [19].

We did not observe any changes in maternal GDF-15 levels in response to anemia or malaria exposure in pregnancy. Endogenous GDF-15 is a ubiquitous cellular stress signal that is produced and secreted by multiple cell types [36]. Mechanistically, it is well documented that food intake and body weight reduction is stimulated via the brainstem-restricted GDF-15 receptor GFRAL (glial cell-derived neurotrophic factor [GDNF] family receptor α-like), and hence GDF-15 and its receptor are highly attractive metabolic disease targets [23]. However, currently only a fraction of the effects mediated by GDF-15 can be explained through the above-mentioned pathway. For example, the physiological role of GDF-15 in pregnancy remains poorly understood. Importantly, the strong predictive value of GDF-15 as biomarker may plausibly be linked to its immune-regulatory function [23]. Therefore, it is noteworthy that malaria exposure did not affect maternal GDF-15 production. This may be explained by the fact that the participants in our study were well treated against malaria, as they were monitored multiple times during pregnancy, and hence were offered treatment immediately when malaria was diagnosed.

### Moderate to Severe Anemia, Particularly in Early Pregnancy, and Malaria Were Associated With Cord Blood FGF-21 Levels in Opposite Directions

Our findings show a clear impact of maternal moderate to severe anemia on cord blood FGF-21 levels. This association was further enforced by correlations showing a negative association between maternal Hb levels in pregnancy and the offspring FGF-21 levels. This result indicates that the effect on maternal anemia is dose dependent. This is supported by the fact that any type of anemia (Hb below 11.0 g/dL) was only modestly associated with offspring FGF-21 levels. In addition, the results show that preconception and early pregnancy anemia (before week 22) is to a higher degree associated with cord blood FGF-21 levels than if the anemia occurred later in pregnancy (after week 22). Importantly, most of the women who had anemia in the third trimester also had anemia in the beginning of the pregnancy. Hence, the anemia exposure on cord blood FGF-21 levels is here suggested also to be duration dependent as well as dose dependent.

It is notable that the described effects on newborn FGF-21 levels are observed despite the fact that the participants in our study were offered preventive treatment for anemia by folic acid and iron supplement, with increased doses when anemia was diagnosed. Hence, it is likely that the consequences of anemia would be even more severe in a non-study setting with limited resources for close monitoring of hemoglobin levels. The impact of maternal anemia on offspring FGF-21 levels needs to be studied in more detail, in longitudinal childhood and adolescence studies, to be able to elucidate the long-term effects on particularly the offspring's cardiometabolic health, especially in settings where anemia is very prevalent and preventive treatment is sparse.

In contrast to anemia, maternal malaria infection in the third trimester was associated with lower cord blood FGF-21 levels and similar patterns were observed in the first and second trimesters, although not statistically significant. The opposing impact of anemia and malaria exposure on FGF-21 levels are in line with previous findings from the same study cohort, where we have estimated the placental vascularization, which was found to be decreased with malaria [13] and increased with anemia [14] in pregnancy. It is also likely that the opposite findings of FGF-21 levels in malaria vs anemia exposure are linked to different biological pathways behind these associations. Whether FGF-21 is a marker, predictor, or regulator of placental formation and vascularization is unknown; therefore, future studies are needed to elucidate this. A potential hypothesis could be that FGF-21 is involved through fetal and/or placental sensing of nutrient supply in order to support adequate nutrition for the fetus through compensatory mechanisms to increase placental vascularity.

In the present study, we also found that anemia in late pregnancy was associated with increased levels of GDF-15 in cord blood. It can be speculated that this is a compensatory mechanism, since GDF-15 has been suggested to suppress hepcidin, a key negative regulator of the entry of iron into the circulation, and thereby increase iron uptake and hemoglobin synthesis [37]. However, other studies suggest that GDF-15 is not necessary for suppression of hepcidin [38]; therefore, further studies are needed to elucidate this.

Regarding the relatively large loss of cord blood sample size due to hemolysis (from *n* = 353 to *n* = 324), this is indeed a limitation of the study; however, unfortunately cord blood hemolysis a mostly unavoidable complication due to the timing of the blood sampling from the cord.

### Association Between Cord Blood FGF-21 and GDF-15 Levels and Neonatal Body Composition

We observed negative associations between both FGF-21 and GDF-15 in cord blood and neonatal body composition. However, our associations were relatively weak and may be interpreted with caution due to the risk of chance findings. However, in the present study all women with anemia and with malaria were offered treatment for the diseases. This may have partially mitigated the influence on GDF-15 and FGF-21 levels among the cases and thereby diminished the ability to see an impact on the neonatal body composition. Our findings of negative correlations between cord blood GDF-15 levels and skinfold thickness of the newborn are supported by another study following 103 infants from birth to 24 months of age, which found that serum GDF-15 levels associated negatively with the change in total fat and abdominal fat, and with changes in ponderal index, throughout the study [39]. Our findings of negative associations between cord blood FGF-21 and neonatal body composition are in contrast to another study that showed positive associations between cord blood FGF-21 and BMI Z-score at 12, 24, and 48 months [20]. FGF-21 is known to promote brown adipose tissue activity and thereby to be protective against obesity through the brown adipose tissue–mediated thermogenic energy expenditure, which could support our finding of a negative association between FGF-21 and skinfold measurements. However, further studies are needed to investigate the role of both cord blood FGF-21 and GDF-15 and their impact on postnatal body composition.

## Conclusions

Moderate to severe anemia, to a higher degree than mild anemia, and particularly at preconception and in the first 22 weeks of pregnancy is associated with 3-fold higher FGF-21 levels in the offspring cord blood. Increased cord blood FGF-21 levels can be speculated to function as a regulatory mechanism to improve placental vascularization and fetal nutrient supply. Malaria exposure in the third trimester pregnancy oppositely results in lower cord FGF-21 levels. Cord blood FGF-21 and GDF-15 levels are negatively associated with neonatal skinfold thicknesses and Z-score, and this may potentially impact the future cardiometabolic health of the child.

## Data Availability

The datasets obtained and analyzed in the current study are available from the first and last authors on reasonable request.

## Abbreviations

BMI: body mass index
FGF-21: fibroblast growth factor 21
FOETALforNCD: Foetal exposure and Epidemiological Transition: the role of Anaemia in early Life for Non-Communicable Diseases in later life
GA: gestational age
GDF-15: growth differentiation factor 15
Hb: hemoglobin
IUGR: intrauterine growth restriction
LBW: low birthweight
LMIC: low- and middle-income countries
MUAC: mid-upper-arm circumference
NCDs: noncommunicable diseases
SSA: sub-Saharan Africa
